# Denosumab Injection Combined with Intramedullary Nail Fixation for Osteoporotic Fractures

**DOI:** 10.1007/s43465-026-01770-w

**Published:** 2026-03-17

**Authors:** Weichu Cai, Changfan Li, Shuoxin Chen, Yijia Gao

**Affiliations:** 1 The First Clinical College of Guangzhou, University of Chinese Medicine, Guangzhou, China; 2 The First Affiliated Hospital of Guangzhou University of Chinese Medicine, Guangzhou, China

**Keywords:** Denosumab, Intramedullary nail fixation, Osteoporotic fractures, Femoral intertrochanteric fracture

## Abstract

**Objective:**

To investigate the clinical efficacy of denosumab injection combined with intramedullary nail fixation for osteoporotic femoral intertrochanteric fractures.

**Methods:**

A total of 106 patients diagnosed with osteoporotic femoral intertrochanteric fractures, classified according to the Evans–Jensen system, who were admitted to The First Affiliated Hospital of Guangzhou University of Chinese Medicine between May 2019 and May 2024. All patients underwent internal fixation using the proximal femoral nail antirotation (PFNA) system. The study group received denosumab injections within 1 week postoperatively, and both groups were administered conventional osteoporosis treatment following surgery. The baseline data (age, sex, cause of injury, and Evans–Jensen classification), operation time, intraoperative blood loss, length of hospital stay, fracture healing time, *T* value of hip bone density, and postoperative complications were compared between these two groups. The VAS score and HHS was evaluated 1 year after surgery.

**Results:**

All 106 patients were followed up for at least 1 year. There was no significant difference in operation time, intraoperative blood loss, hospital stays, fracture healing time, and HHS between the two groups (*P* > 0.05). The incidence of postoperative complications, *T* value of hip bone density, and VAS score in the study group was significantly lower than that in the control group, and the difference between the groups was statistically significant (*P* < 0.05).

**Conclusion:**

In summary, the integrative therapeutic protocol combining denosumab administration with proximal femoral nail antirotation (PFNA) fixation demonstrates marked efficacy in enhancing femoral neck bone mineral density and reducing fracture-related limb pain among elderly patients with osteoporotic intertrochanteric fractures. This evidence-based dual-intervention strategy not only optimizes fracture healing outcomes, but also represents a valuable clinical approach for comprehensive postoperative rehabilitation in this vulnerable patient population.

## Introduction

Denosumab, a humanized monoclonal antibody targeting the receptor activator of nuclear factor kappa-B ligand (RANKL), inhibits osteoclast formation and activity by reducing bone resorption [1], making it a cornerstone in osteoporosis treatment. Clinical studies confirm its efficacy in increasing bone mineral density (BMD) and lowering fracture risk in postmenopausal osteoporosis (PMOP) [2, 3]. PMOP patients treated with denosumab for 6 months showed significant increases in lumbar spine and femoral neck BMD, reductions in bone turnover markers such as C-terminal telopeptide of type I collagen (CTX), and procollagen type I N-terminal propeptide (P1NP), with a low adverse event rate (2.5%) and no delayed or nonunion fractures [4, 5]. Compared to bisphosphonates, denosumab’s reversible mechanism avoids bone matrix deposition, but requires monitoring for hypocalcemia and osteonecrosis of the jaw [6, 7]. Furthermore, recently study indicated denosumab’s superiority over alendronate in reducing major osteoporotic fracture (MOP) risk by 31% and hip fracture risk by 36% with 5-year treatment [8].

Recent research highlights denosumab’s potential in osteoporotic fracture healing. Preclinical studies suggest that RANKL inhibition reduces early-stage osteoclast overactivation, promoting callus mineralization and mechanical strength. Clinical observations show improved healing quality in elderly patients with hip or vertebral fractures, potentially reducing secondary fractures by preserving bone microstructure. A randomized trial found denosumab superior to zoledronic acid in callus volume and biomechanical strength at 12 weeks post-wrist fracture [9]. However, optimal timing remains unclear. Denosumab, as a core anti-osteoporosis drug, combined with novel biomaterials, accelerates fracture repair [10, 11]. Future studies should focus on its molecular mechanisms and synergies with other therapies.

Advances in femoral intertrochanteric fracture surgery include minimally invasive techniques and postoperative rehabilitation. For elderly patients with unstable fractures and osteoporosis, hemiarthroplasty enables early weight-bearing, reduces complications, and restores hip function rapidly, with an 88.6% long-term success rate. PFNA (proximal femoral nail antirotation) combined with ERAS (enhanced recovery after surgery) allows ambulation within 24 h [12]. Surgical timing within 4 days post-injury shows similar complication risks to early (24 h) intervention, provided preoperative optimization is performed. Improved intramedullary nails and plates enhance stability, but severe osteoporosis or fixation failures favor arthroplasty [13]. However, no studies have explored the combined use of PFNA with denosumab or other anti-osteoporosis drugs for fracture healing, requiring further research.

Thus, the purpose of this study was to compare the clinical efficacy of denosumab injection combined with intramedullary nail fixation for osteoporotic femoral intertrochanteric fractures.

## Methods and Materials

### Patients

105 patients diagnosed with femoral intertrochanteric fractures were admitted to The First Affiliated Hospital of Guangzhou University of Chinese Medicine between May 2019 and May 2024. Patients in the study group were screened for contraindications to denosumab, including pre-existing hypocalcemia (corrected serum calcium < 8.5 mg/dL), known hypersensitivity to denosumab or its components, and pregnancy/lactation. All eligible patients provided written informed consent specifically for denosumab use, acknowledging the potential risks including hypocalcemia, osteonecrosis of the jaw, and atypical femoral fractures, although these risks are low with short-term use.

All procedures performed in this study were in accordance with the ethical standards of the institutional review board.

### Medicine Intervention

In the study group, denosumab (Amgen, USA) was administered as a single subcutaneous injection of 60 mg within 1 week postoperatively (mean: 5.2 ± 1.4 days; range: 3–7 days). The 60 mg dose is the standard approved regimen for osteoporosis treatment, independent of patient weight or age. To prevent denosumab-associated hypocalcemia and optimize bone health, all patients in both groups received standardized calcium and vitamin D supplementation throughout the follow-up period. Specifically, patients were prescribed daily oral calcium carbonate (equivalent to 1000 mg elemental calcium) and vitamin D_3_ (800 IU), commencing on the day of denosumab administration (or at discharge for the control group) and continuing for the entire 12-month study duration. Compliance was reinforced during each follow-up visit.

The control group received conventional osteoporosis treatment consisting of the same calcium and vitamin D supplementation, without antiresorptive or anabolic agents during the 12-month follow-up period. This study design allowed for evaluation of the additive effect of denosumab on standard postoperative osteoporosis management.

### Inclusion and Exclusion Criteria

The inclusion criteria were as follows. (1) Femoral intertrochanteric fractures. (2) Osteoporosis was confirmed by preoperative dual-energy X-ray absorptiometry (DXA) with a T-score ≤ − 2.5 at the femoral neck or lumbar spine, in accordance with WHO criteria. Based on the T-score, osteoporosis severity was further categorized as moderate (− 2.5 > T ≥ -3.0) or severe (T < − 3.0). (3) Follow-up of at least 12 months.

The exclusion criteria were as follows: (1) local or systemic infection; (2) patients with hematological diseases; (3) multiple fractures.

### Surgery Procedure

All fractures were classified preoperatively using the Evans–Jensen system to assess fracture geometry and stability. All patients underwent fixation with the proximal femoral nail antirotation (PFNA, Synthes, Switzerland), a standardized intramedullary device designed for unstable trochanteric fractures. The patient was positioned supine on a traction table under lumbar or general anesthesia. Closed reduction was performed via traction to restore the femoral neck–shaft angle and correct rotational deformity. A 3–5 cm longitudinal incision was made proximal to the tip of the greater trochanter. Under fluoroscopy guidance, the entry point was carefully localized slightly medial to the trochanteric apex in the anteroposterior view and aligned with the medullary canal axis in the lateral view. A guidewire was inserted into the medullary cavity, followed by sequential reaming according to preoperative planning. The appropriately sized PFNA main nail was then advanced along the guidewire, with intraoperative fluoroscopy confirming its optimal position: 5–10 mm below the femoral head on anteroposterior projection and centered within the femoral neck on the lateral projection.

Subsequently, a guidewire was directed into the femoral neck through the targeting device, terminating 5–10 mm from the articular surface. After depth measurement, the helical blade was inserted into the subchondral bone by rotational impaction to enhance compression and rotational stability. Distal locking was achieved using a static or dynamic screw through the targeting system, followed by proximal tail cap placement to seal the nail. Postoperative radiographs verified satisfactory fracture reduction and implant positioning. Early non-weight-bearing mobilization was initiated within 24–48 h, with progressive weight-bearing guided by fracture consolidation. Critical intraoperative considerations included avoiding lateral deviation of the entry point to prevent varus malalignment, minimizing bone drilling in osteoporotic patients, and implementing postoperative thromboprophylaxis with anticoagulants alongside antibiotic coverage.

### Perioperative Assessment

Perioperative outcomes were prospectively recorded, including: operative time (minutes), estimated intraoperative blood loss (mL), radiographic fracture union time, and total hospital stay duration (days).

### Postoperative Evaluation

Postoperative assessments comprised: 1) bone mineral density (BMD, T-score measured via dual-energy X-ray absorptiometry (DXA)); 2) patient-reported satisfaction using a ten-point visual analog scale (VAS); 3) functional recovery quantified by the Harris Hip Score (HHS); and 4) complication (surgical site infection, femoral shortening > 1 cm, nonunion at 6 months, avascular necrosis of the femoral head, implant removal, and revision surgery for mechanical failure).

### Data Statistics

Continuous variables were expressed as mean ± standard deviation for normally distributed data or median (interquartile range) for skewed distributions. Intergroup comparisons of preoperative baseline characteristics were performed using the Wilcoxon rank-sum test for nonparametric continuous variables and Pearson’s *χ*^2^ test (or Fisher's exact test when expected cell counts < 5) for categorical variables. Statistical significance was defined as a two-tailed *P* < 0.05. All analyses were implemented using IBM SPSS Statistics version 20.0.

## Results

### Characteristics of Patients

All patients were followed up for at least 1 year with X-ray film obtained at postoperative 1, 6, and 12 months. Fifty patients treated with denosumab injection combined with PFNA (study group) and 56 patients treated with cannulated compression screw (control group) were included in our study. The average age was 62.17 ± 2.58 years in the study group and 60.17 ± 3.42 years in the control group. Twenty-four patients were male in the study group and 26 in the control group. Traffic injury was the main cause of injury in both groups. Fracture geometry and stability were categorized according to the Evans–Jensen classification. There were 6 patients with Type I, 15 patients with Type II, 20 patients with Type III, and 9 patients with Type IV in the study group and 4 patients with Type I, 17 patients with Type II, 27 patients with Type III, and 8 patients with Type IV in the control group. The baseline data of both groups are shown in Table 1, which demonstrates that the patients in the distribution of each characteristic were similar in the two groups.

**Table 1:** Characteristics of patients

	Study group (*n* = 50)	Control group (*n* = 56)	*P* value
Gender, *n* (%)			
Female	26 (52.0)	30 (53.6)	0.88
Male	24 (48.0)	26 (46.4)	
Age [years]	62.17 ± 2.58	60.17 ± 3.42	0.76
Cause of injury, *n* (%)			0.65
Traffic injury	22 (44.0)	25 (44.6)	
High falling	18 (36.0)	17 (30.4)	
Crushing	10 (20.0)	14 (25.0)	
Evans–Jensen classification, *n* (%)			0.44
Type I	6 (12.0)	4 (7.1)	
Type II	15 (30.0)	17 (30.4)	
Type III	20 (40.0)	27 (48.2)	
Type IV	9 (18.0)	8	
Preoperative T-score (mean ± SD)	− 3.01 ± 0.33	− 3.12 ± 0.41	0.86
Osteoporosis severity			0.92
Moderate (− 2.5 > T ≥ − 3.0)	18	21	
Severe (T < − 3.0)	32	35	

*Abbreviations: SD, standard deviation*

**Table 2:** Surgical outcomes in these two groups

	Study group	Control group	*P* value
Surgical time (min)	75.82 ± 20.20	70.82 ± 22.60	0.56
Surgical blood loss (ml)	48.80 ± 5.45	50.60 ± 4.86	0.88
Length of hospital stay (day)	7.55 ± 1.23	7.86 ± 1.08	0.87
VAS score 1 year postoperatively	2.85 ± 0.52	4.85 ± 0.50	< 0.01
Harris hip score (HHS) 1 year postoperatively	91.42 ± 5.26	92.28 ± 4.88	0.67
Fracture healing time (months)	11.44 ± 1.82	14.26 ± 2.66	< 0.01

In the study group, 32 patients (64%) had severe osteoporosis (T < − 3.0) and 18 (36%) had moderate osteoporosis (− 2.5 > T ≥ − 3.0); in the control group, 35 patients (62.5%) had severe and 21 (37.5%) had moderate osteoporosis. No significant difference was observed in the distribution of osteoporosis severity (*P* = 0.92).

### Clinical Data Analysis

The mean operation times were 75.82 ± 20.20 min in the study group and 70.82 ± 22.60 min in the control group (*P* = 0.56). The mean blood loss was 48.80 ± 5.45 ml in the study group and 50.60 ± 4.86 ml in the control group (*P* = 0.88). The mean hospital stays was 7.55 ± 1.23 days in the study group and 7.86 ± 1.08 days in the control group (*P* = 0.87). The VAS score 1 year postoperatively was lower in the study group (2.85 ± 0.52 vs. 4.85 ± 0.50, *P* < 0.01). The HHS was 91.42 ± 5.26 in the study group and 92.28 ± 4.88 in the control group (*P* = 0.67). Fracture healing time was 11.44 ± 1.82 months in the study group and 14.26 ± 2.66 in the control group (*P* < 0.01) (Table 2). Furthermore, the T value of hip bone density was −3.01 ± 0.33 in the study group and − 3.12 ± 0.41 in the control group before surgery (*P* = 0.86). One year postoperatively, the T value of hip bone density was − 2.04 ± 0.20 in the study group and − 2.55 ± 0.31 in the control group (*P* < 0.01) (Table 3). Moreover, the complication rates were 6% (3/50) in the study group and 17.86% (10/56) in the control group (*P* < 0.01) (Table 4).

**Table 3:** Comparison of *T* value between these two groups

	Study group	Control group	*P* value
Preoperative	− 3.01 ± 0.33	− 3.12 ± 0.41	0.86
1 year postoperative	− 2.04 ± 0.20	− 2.55 ± 0.31	< 0.01

**Table 4:** Complications in these two groups

	Study group	Control group	*P* value
Complications			< 0.01
Nonunion	0	0	
Femoral head necrosis	2	6	
Nail withdrawal	1	3	
Cut out the internal fixation	0	1	

## Discussion

The management of osteoporotic intertrochanteric fractures necessitates a dual-focused approach encompassing both anatomical reconstruction and metabolic bone optimization. While surgical intervention remains the cornerstone of treatment, the selection of appropriate modalities requires nuanced consideration. The proximal femoral nail antirotation (PFNA) system has emerged as the gold standard for fragility fractures in geriatric populations, with its helical blade design demonstrating superior rotational stability and trabecular bone compaction capabilities in osteoporotic medullary cavities [14]. In cases of severe comminution or unstable fracture patterns, arthroplasty procedures may offer accelerated mobility recovery, albeit at the expense of higher perioperative risks and socioeconomic burden. Pharmacotherapeutic challenges persist, particularly regarding bisphosphonate administration protocols—premature discontinuation before the recommended 3- to 5-year therapeutic window correlates with suppressed bone remodeling and atypical subtrochanteric fractures.

In the prevention and treatment of osteoporotic fractures, the study further revealed that disumab can create a favorable microenvironment for bone repair by inhibiting abnormal bone resorption after fracture. The degree of osteoporosis, as reflected by baseline T-scores, is a critical determinant of fracture healing and implant stability in patients with intertrochanteric fractures. In our cohort, the majority of patients presented with severe osteoporosis (T < − 3.0), which is consistent with the typical fragility fracture population. Importantly, the comparable distribution of osteoporosis severity between the two groups ensures that the observed benefits of denosumab—namely improved BMD and reduced pain—are not confounded by baseline imbalances. Previous studies have suggested that patients with more severe osteoporosis may derive greater absolute benefit from potent antiresorptive therapy like denosumab, although our sample size precludes subgroup analysis. Future research should explore whether the efficacy of combined PFNA and denosumab varies by osteoporosis severity.

The inclusion of both radiological and functional outcomes in this study reflects the contemporary understanding that successful fracture management extends beyond bony union to encompass patient-centered endpoints. While the significant improvement in femoral neck BMD (from − 3.01 ± 0.33 to − 2.04 ± 0.20, *P* < 0.01) confirms the expected pharmacological effect of denosumab, the accompanying reduction in VAS pain scores (2.85 ± 0.52 vs. 4.85 ± 0.50, *P* < 0.01) demonstrates that this bone density benefit translates into tangible clinical improvement. Importantly, comparable HHS between groups (91.42 ± 5.26 vs. 92.28 ± 4.88, *P* = 0.67) indicates that denosumab does not impede functional recovery—a critical safety consideration given concerns about potential over-suppression of bone remodeling. Similar surgical parameters (operative time, blood loss, hospital stay) between groups further confirm that early postoperative denosumab administration is safe and does not complicate the surgical course. Thus, this multidimensional evaluation strengthens the clinical applicability of our findings: denosumab not only improves bone health, but also enhances the quality of recovery by reducing pain without compromising surgical outcomes or functional restoration.

## Conclusion

In conclusion, the combined use of denosumab and proximal femoral nail antirotation demonstrates significant efficacy in improving hip bone mineral density and alleviating limb pain in osteoporotic intertrochanteric fractures, establishing this dual-modality approach as a clinically valuable therapeutic strategy for postoperative management.

## Data Availability

The datasets generated and/or analyzed during the current study are not publicly available due to patient privacy, but are available from the corresponding author upon reasonable request.

## References

[1] Shane, E., Shiau, S., Recker, R. R., Lappe, J. M., Agarwal, S., Kamanda-Kosseh, M., Bucovsky, M., Stubby, J., & Cohen, A. (2022). Denosumab after Teriparatide in premenopausal women with idiopathic osteoporosis. *Journal of Clinical Endocrinology and Metabolism,**107*(4), e1528–e1540. 10.1210/clinem/dgab850 PMC9122662 34849989

[2] Cosman, F., Crittenden, D. B., Ferrari, S., Khan, A., Lane, N. E., Lippuner, K., Matsumoto, T., Milmont, C. E., Libanati, C., & Grauer, A. (2018). FRAME Study: The foundation effect of building bone with 1 year of Romosozumab leads to continued lower fracture risk after transition to Denosumab. *Journal of Bone and Mineral Research,**33*(7), 1219–1226. 10.1002/jbmr.3427 29573473

[3] Bone, H. G., Wagman, R. B., Brandi, M. L., Brown, J. P., Chapurlat, R., Cummings, S. R., Czerwinski, E., Fahrleitner-Pammer, A., Kendler, D. L., Lippuner, K., et al. (2017). 10 years of denosumab treatment in postmenopausal women with osteoporosis: Results from the phase 3 randomised FREEDOM trial and open-label extension. *The Lancet. Diabetes & Endocrinology,**5*(7), 513–523. 10.1016/S2213-8587(17)30138-9 28546097

[4] Miller, P. D., Pannacciulli, N., Malouf-Sierra, J., Singer, A., Czerwinski, E., Bone, H. G., Wang, C., Huang, S., Chines, A., Lems, W., et al. (2020). Efficacy and safety of denosumab vs. bisphosphonates in postmenopausal women previously treated with oral bisphosphonates. *Osteoporosis International,**31*(1), 181–191. 10.1007/s00198-019-05233-x 31776637

[5] Grassi, G., Ghielmetti, A., Zampogna, M., Chiodini, I., Arosio, M., Mantovani, G., & Eller-Vainicher, C. (2024). Zoledronate after Denosumab discontinuation: Is repeated administrations more effective than single infusion? *Journal of Clinical Endocrinology and Metabolism,**109*(10), e1817–e1826. 10.1210/clinem/dgae224 PMC11403318 38609157

[6] Everts-Graber, J., Lehmann, D., Burkard, J. P., Schaller, B., Gahl, B., Hauselmann, H., Studer, U., Ziswiler, H. R., Reichenbach, S., & Lehmann, T. (2022). Risk of osteonecrosis of the jaw under Denosumab compared to bisphosphonates in patients with osteoporosis. *Journal of Bone and Mineral Research,**37*(2), 340–348. 10.1002/jbmr.4472 34787342

[7] Khan, A. A., Morrison, A., Hanley, D. A., Felsenberg, D., McCauley, L. K., O’Ryan, F., Reid, I. R., Ruggiero, S. L., Taguchi, A., Tetradis, S., et al. (2015). Diagnosis and management of osteonecrosis of the jaw: A systematic review and international consensus. *Journal of Bone and Mineral Research,**30*(1), 3–23. 10.1002/jbmr.2405 25414052

[8] Kendler, D. L., Cosman, F., Stad, R. K., & Ferrari, S. (2022). Denosumab in the treatment of osteoporosis: 10 years later: A narrative review. *Advances in Therapy,**39*(1), 58–74. 10.1007/s12325-021-01936-y PMC8799550 34762286

[9] Fizazi, K., Carducci, M., Smith, M., Damiao, R., Brown, J., Karsh, L., Milecki, P., Shore, N., Rader, M., Wang, H., et al. (2011). Denosumab versus zoledronic acid for treatment of bone metastases in men with castration-resistant prostate cancer: A randomised, double-blind study. *Lancet,**377*(9768), 813–822. 10.1016/S0140-6736(10)62344-6 PMC3090685 21353695

[10] Kim, S. J., Kim, J. W., & Lee, D. W. (2022). Denosumab versus zoledronic acid in elderly patients after hip fracture. *Journal of Orthopaedic Surgery (Hong Kong),**30*(3), 10225536221147082. 10.1177/10225536221147082 36541833

[11] Gogakos, A. I., & Anastasilakis, A. D. (2025). Current and emerging bone resorption inhibitors for the treatment of osteoporosis. *Expert Opinion on Pharmacotherapy,**26*(3), 265–278. 10.1080/14656566.2025.2451741 39797385

[12] Kang, Y., Liu, J., Chen, H., Ding, W., Chen, J., Zhao, B., & Yin, X. (2019). Enhanced recovery after surgery (ERAS) in elective intertrochanteric fracture patients result in reduced length of hospital stay (LOS) without compromising functional outcome. *Journal of Orthopaedic Surgery and Research,**14*(1), 209. 10.1186/s13018-019-1238-2 PMC6617739 31288824

[13] Murugan, P. B., Mohideen, S., Pradeep, E., Kumar, K. V. A., Pandian, H., & Ashwin, V. Y. (2024). Comparison of functional and radiological outcome of unstable intertrochanteric femur fractures treated using PFN and PFNA in patients with osteoporosis. *Journal of Orthopaedic Case Reports,**14*(10), 219–224. 10.13107/jocr.2024.v14.i10.4870 PMC11458224 39381321

[14] Neuerburg, C., Mehaffey, S., Gosch, M., Bocker, W., Blauth, M., & Kammerlander, C. (2016). Trochanteric fragility fractures : Treatment using the cement-augmented proximal femoral nail antirotation. *Operative Orthopaedics and Traumatology,**28*(3), 164–176. 10.1007/s00064-016-0449-5 27245659

